# Antiarrhythmic potentials of irisin in ischemia/reperfusion injury of diabetic rats through modulating mitochondria-endoplasmic reticulum interaction and inhibiting pyroptosis

**DOI:** 10.22038/ijbms.2024.78069.16878

**Published:** 2024

**Authors:** Xiaona Zhang Zhang, Kai Jing, Wei Ma, Jin Wang

**Affiliations:** 1 Department of Cardiovascular Diseases, Xi’an International Medical Center Hospital, Xi’an, 710100, China; 2 Department of Proctology, The People’s Hospital of Huaiyin Jinan, 250021, Shandong, China; 3 Department of Cardiology, The Fifth People’s Hospital of Jinan, 250022, Shandong, China

**Keywords:** Arrhythmia Cardioprotection, Diabetes, Inflammation, Pyroptosis, Reperfusion

## Abstract

**Objective(s):**

Myocardial arrhythmia is a major complication of ischemia-reperfusion (I/R) injury in patients with diabetes. Irisin has significant cardioprotective effects, while its role in the pathophysiology of I/R injury-induced myocardial arrhythmia in the presence of diabetes is not well identified. Here, we aimed to investigate the potential antiarrhythmic impacts and mechanisms (mitochondrial biogenesis, endoplasmic reticulum (ER) stress, and pyroptosis) by which irisin reduces I/R injury-induced myocardial arrhythmia in diabetic rats.

**Materials and Methods:**

Thirty high-fat diet-induced diabetic rats were subjected to I/R injury and myocardial arrhythmia. Irisin (0.5 μg/kg/day) was injected intraperitoneally before induction of I/R injury. Electrocardiography was used to measure the incidence and severity of ventricular arrhythmias. ELISA and western blotting analyses were employed to quantify the expression of mitochondrial biogenesis, ER stress, and pyroptosis-related proteins in ischemic myocardium.

**Results:**

Irisin treatment in diabetic rats significantly decreased the lactate dehydrogenase level and the number and severity of arrhythmia induced by I/R injury. Irisin up-regulated the expression of mitochondrial biogenesis-related proteins while down-regulating the expression of ER stress and pyroptosis-related proteins. Furthermore, the inhibition of mitochondrial quality control by mdivi-1 significantly abolished the cardioprotective effect of irisin.

**Conclusion:**

Our findings suggest that irisin reduced myocardial arrhythmia induced by I/R injury in diabetic rats by modulating the interaction of mitochondrial biogenesis and ER stress proteins and inhibiting the pyroptosis pathway. These findings provide a promising strategy for managing myocardial arrhythmia in diabetic patients, but supplementary studies are needed to confirm the clinical efficacy of irisin in these patients.

## Introduction

The clinical significance of arrhythmia following myocardial ischemia/reperfusion (I/R) injury lies in its pivotal role as a prognostic indicator and a potential therapeutic target in cardiovascular medicine. Myocardial arrhythmia, characterized by abnormal heart rhythms, is a significant consequence of I/R injury in diabetic patients following percutaneous coronary intervention, coronary artery bypass grafting, and heart transplantation (1, 2). The development of arrhythmia during I/R injury in the presence of diabetes is intricate and involves a multitude of molecular and cellular mechanisms, contributing to its complex pathogenesis (2). Effective management of arrhythmias in diabetic subjects post-IR not only addresses the immediate cardiac concerns but also potentially mitigates long-term cardiovascular complications, significantly reducing morbidity and mortality. 

Pyroptosis, endoplasmic reticulum (ER) stress, and mitochondrial dysfunction are three key factors that may contribute to I/R injury-induced myocardial dysfunction and arrhythmia in diabetic conditions (1,3). Pyroptosis is a type of programmed cell death that is induced by inflammasomes, which are large protein complexes that induce caspase-1 activation and prompt the release of proinflammatory cytokines, including interleukin-1 beta (IL-1β) and IL-18 (4). It has been shown that pyroptosis was significantly increased in the hearts of diabetic rats suffering from I/R injury (5). Pharmacological inhibition of pyroptosis attenuated myocardial I/R injury. It boosted heart hemodynamics in diabetic rats, suggesting that targeting pyroptosis can be a hopeful therapeutic strategy for reducing I/R injury outcomes in diabetic conditions. 

Endoplasmic reticulum (ER) stress is a cellular response to various stressors, such as hypoxia and nutrient deprivation, that disrupts the proper folding and processing of proteins in the ER (6). However, severe and prolonged ER stress may cause cell death, including apoptosis and necroptosis (7). In the context of myocardial I/R injury in diabetes, ER stress is a key mechanism that contributes to the exacerbation of oxidative stress, inflammation, and apoptosis following mitochondrial dysfunction (8). CHOP (C/EBP homologous protein) and eIF-2α (eukaryotic translation initiation factor 2 alpha) are pivotal regulators of the cellular response to ER stress (6, 7). ER stress triggers the activation of ER kinases, leading to the phosphorylation of eIF-2α. Phosphorylated eIF-2α attenuates global protein synthesis while promoting the translation of activating transcription factor 4 (ATF4). ATF4 induces the expression of CHOP, amplifying the cellular response to ER stress. Sustained CHOP expression can lead to excessive apoptosis and cell death under prolonged ER stress in diabetic conditions (6). 

Mitochondrial biogenesis involves the formation of new mitochondria and remodeling existing ones, which is critical for maintaining mitochondrial quality control and energy metabolism following reperfusion therapy (9). Mitochondrion plays an empirical role in cellular metabolism and energy production, and mitochondrial dysfunction is a hallmark of I/R injury in diabetes, which impairs oxidative phosphorylation and ATP production and contributes to cell death (10). Thus, a mitochondrial quality control system is a potential therapeutic target for diabetic cardiomyopathy, as it may restore mitochondrial function and prevent I/R injury. PGC-1α (peroxisome proliferator-activated receptor gamma coactivator-1 alpha) and Sirtuin 3 (Sirt3) are pivotal regulators of mitochondrial activity and biogenesis, exerting profound effects on cellular metabolism and energy homeostasis (10). PGC-1α acts as a transcriptional coactivator, promoting the expression of genes involved in mitochondrial biogenesis, metabolism, and respiratory chain function, thus enhancing mitochondrial synthesis and oxidative capacity. Meanwhile, Sirt3, a mitochondrial deacetylase, modulates mitochondrial function by deacetylating key metabolic enzymes and transcription factors, promoting mitochondrial biogenesis and improving oxidative phosphorylation efficiency (9, 10). 

Irisin is a myokine produced in skeletal muscles and secreted into circulation. It is known to have beneficial effects on energy metabolism and glucose homeostasis (11, 12). Recently, irisin has emerged as a potential therapeutic agent for various diseases, such as diabetes, metabolic syndrome, and cardiovascular diseases (11). It had a protective effect on the heart with I/R injury by improving cardiac function, reducing inflammation, and promoting angiogenesis (12). Recent studies found that irisin treatment also significantly reduced microvascular injury and the outcomes of I/R injury in diabetic hearts (13). It has also been found that irisin can recover mitochondrial function and lessen oxidative stress, which may contribute to the cardioprotective potentials of irisin upon I/R injury (14). This agent possesses significant anti-inflammatory impacts and may inhibit the pyroptosis pathway (15). 

Collectively, it can be inferred that irisin holds promise as a treatment option for decreasing myocardial arrhythmia triggered by I/R injury in subjects with diabetes. Nonetheless, it is not yet well understood how and by which mechanism this protective agent may reduce the incidence of myocardial arrhythmia in diabetic hearts following I/R injury. Thus, the specific aim of this study was to investigate the potential antiarrhythmic feature of irisin in diabetic rats with I/R injury and to disclose the role of mitochondrial biogenesis, pyroptosis pathway, and ER stress in modulating the effect of irisin in this condition. 

## Materials and methods

***Animal handling, induction of diabetes, and ethics statement***

Thirty male Sprague-Dawley rats (weighing 250-300 g and aged 6-8 weeks) were supplied and housed in a climate-controlled room with a 12-hour light-dark cycle and given unrestricted access to food and water. Following two weeks of adaptation period for rats, diabetes was induced by a high-fat diet (62% calories from fat) and single intraperitoneal injection of streptozotocin (35 mg/kg, citrated buffer; pH 4.5; Sigma Aldrich, USA) after four weeks of feeding high-fat diet. After 72 hr of administering streptozotocin, the rats’ blood glucose levels were assessed, and those with levels equal to or greater than 16.7 mmol/l were classified as diabetic. The diabetic regimen lasted for eight weeks. Every activity involving the use of animals followed the guidelines specified in the Guide for Care and Use of Laboratory Animals (revised in 1996, United States National Institutes of Health Publication No. 85-23) and received approval from the Institutional Ethics Committee.

***Experimental design***

Thirty diabetic animals were divided into five groups at random, with each group consisting of six rats: Sham-operated group (Sham), Ischemia/reperfusion injury group (IR), I/R plus irisin treatment group (IR+Irsn), I/R plus mitochondrial division inhibitor group (I/R+Mdv), and I/R plus mitochondrial division inhibitor group and irisin (I/R+Mdv+Irsn). Sham group experienced the same procedure, but without resulting in any tissue damage. Irisin (Sigma-Aldrich, USA) (0.5 μg/kg/day)(16) was administered to diabetic rats via intraperitoneal injection once daily for seven consecutive days before the surgery. The mitochondrial division inhibitor-1 (mdivi-1, 10 mg/kg/day)(17) (Sigma-Aldrich, USA) was administered intraperitoneally to rats before irisin injections. The rats in the Sham and IR groups were given an equivalent amount of saline. 

***Sample size calculation***

We considered an alpha level of significance at 0.05 and a power of 0.80 to calculate the sample size, ensuring our study’s ability to detect statistically significant differences. Based on previous literature and expected effect sizes, we conducted a power analysis, which indicated that a sample size of six rats per group would provide sufficient statistical power to detect significant differences between the groups.

***I/R injury modeling***

To establish the I/R model, the procedure was conducted in accordance with the previously reported method (2). In brief, the rats were anesthetized with 1% sodium pentobarbital (40 mg/kg) and underwent a left thoracotomy to expose their hearts. The left anterior descending coronary artery was tied off for 30 min, followed by 120 min of reperfusion to create the I/R model. The Sham group underwent an identical procedure but without ligation of the coronary artery.

***Electrocardiogram recording and analysis***

The heart rhythm was monitored using a surface electrocardiogram (ECG) throughout the experiment. Electrocardiograms were recorded during the first 30 min of the ischemic phase and immediately after the initial 30 min of the reperfusion phase. The electrocardiograms were analyzed for the number of premature ventricular complexes (PVC), ventricular tachycardia (VT), and fibrillation (VF) based on the Lambeth convention for arrhythmia interpretation in animals. Additionally, the severity of arrhythmias was scored according to a 5-degree rating system as follows: 0: no arrhythmia; 1: occurrence of ventricular premature beets; 2: occurrence of ventricular bigeminy or salvos; 3: occurrence of VT; and 4: occurrence of VF.

***Measurement of injury marker lactate dehydrogenase***

After the reperfusion period was completed, the rats were anesthetized using 40 mg/kg sodium pentobarbital, and their hearts were exposed. Blood samples were then collected directly from the heart and used to measure lactate dehydrogenase (LDH) levels with an ELISA kit (Nanjing Jiancheng Bioengineering Institute, Nanjing, China). The samples’ absorbance was detected at 340 nm, and the LDH levels were expressed in U/l.

***Measurement of pyroptosis markers***

The levels of the nucleotide-binding domain, leucine-rich containing family, pyrin domain containing 3 (NLRP3), IL-1β (by ELISA), and cleaved caspase-1 (by western blotting) were measured to estimate the levels of pyroptosis in this study. The hearts were harvested after 120 min of reperfusion, and the ischemic regions of the left ventricles were homogenized in RIPA lysis buffer. The levels of NLRP3 and IL-1β were assayed using commercial ELISA kits according to the manufacturer’s instructions (MyBioSource, Inc., USA). The samples’ protein concentration was quantified using the bicinchoninic acid assay kit. The levels of NLRP3 and IL-1β in each sample were normalized according to the protein concentration in the corresponding sample.

***Western blotting***

The expression levels of protein-cleaved caspase-1, PGC-1α, CHOP, and the phosphorylated forms of Sirt3 and eIF-2α were assessed using western blotting. Initially, equal amounts of protein extracted from the left ventricles were loaded onto sodium dodecyl sulfate-polyacrylamide gels and subjected to electrophoresis to separate the proteins. Subsequently, the separated proteins were transferred onto PVDF membranes (Sigma-Aldrich, USA). After blocking the membranes with low-fat milk in Tris-buffered saline pH 7.6 and 0.1% Tween 20, and subsequent washing stages to remove any unbound proteins, primary antibodies specific to the proteins of interest, including β-actin as a loading control (1:1500, Cell Signaling Technology, USA), were then applied to the membranes. Following an incubation period allowing for antibody-antigen binding, the membranes were thoroughly washed to remove any unbound antibodies. Next, secondary antibodies conjugated to horseradish peroxidase were added to the membranes to recognize and bind to the primary antibodies. Finally, the protein bands were visualized using an enhanced chemiluminescence kit (Thermo Fisher Scientific, USA), enabling the detection and quantification of the target proteins. 

***Statistical analysis***

Statistical analysis was carried out utilizing GraphPad Prism 7 software (San Diego, USA). Group differences were assessed employing one-way ANOVA followed by Tukey’s *post-hoc* test. Shapiro-Wilk and Kolmogorov-Smirnov tests were conducted to verify the dataset’s normality across groups. A significance level of *P*<0.05 was considered statistically significant. Full tables containing detailed statistical analysis information were included in a supplementary file.

## Results

***Irisin reduces myocardial arrhythmia induced by I/R injury in diabetic rats***

**Figure 1:**
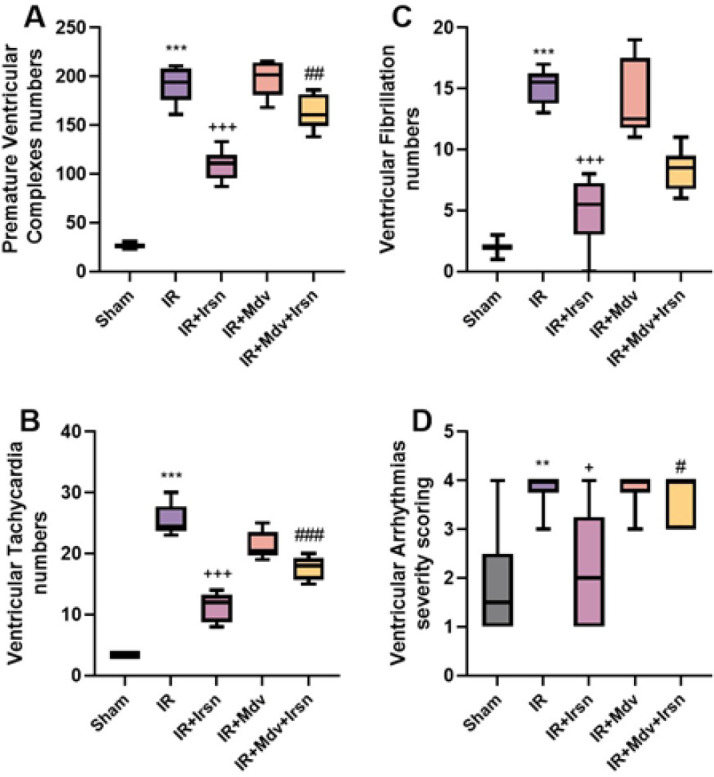
(A-D) Irisin reduced the numbers and severity scoring of ventricular arrhythmias following I/R injury in diabetic rats

To examine the impact of irisin on myocardial arrhythmia caused by I/R injury in diabetic rats, we performed electrocardiogram (ECG) analysis. One-way ANOVA and Tukey tests indicated that the number of PVCs, VT, and VF were significantly increased in the diabetic rats after induction of I/R injury, compared to the sham group (*P*<0.001 for all). Conversely, irisin treatment significantly reduced the occurrence of myocardial arrhythmias in diabetic rats compared to the IR group (*P*<0.001 for all). Additionally, blocking of mitochondrial division through mdivi-1 significantly reduced the protective effects of irisin on IR-induced PVCs (*P*=0.004) and VT (*P*<0.001)(Figure 1A-C). Likewise, induction of I/R injury led to a significant increase in the severity scoring of ventricular arrhythmias compared to that of the sham group (*P*=0.002). This scoring was considerably lower in the irisin-treated group compared to the IR group (*P*=0.013), and this effect was also abolished after the administration of mdivi-1 (*P*=0.029)(Figure 1D). 

***Irisin ameliorates myocardial injury induced by I/R injury in diabetic rats***

**Figure 2:**
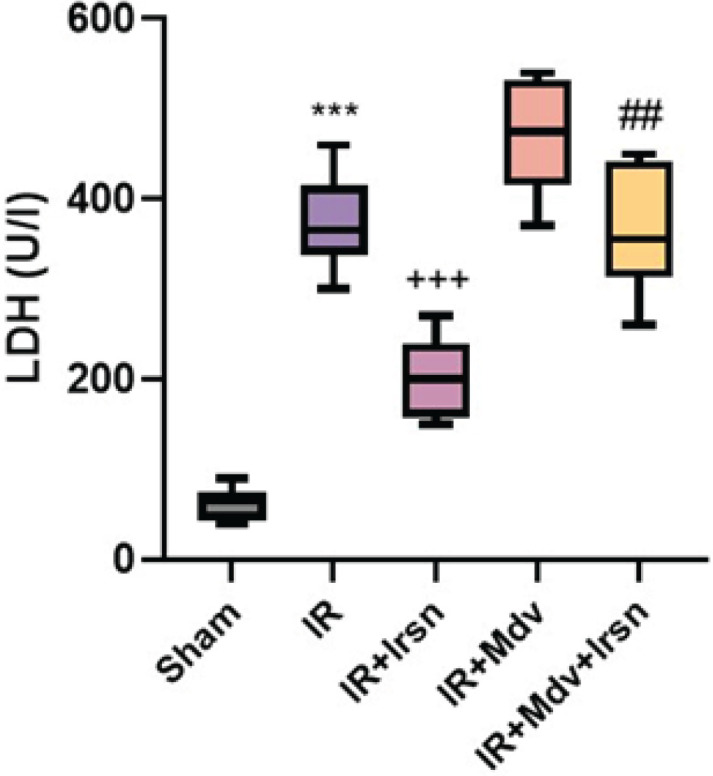
Irisin decreased myocardial injury marker (LDH) following I/R injury in diabetic rats

To further confirm the protective effect of irisin against I/R injury in diabetic rats, we evaluated myocardial injury by measuring the levels of LDH in serum and analyzing the data using one-way ANOVA (Figure 2). We found that the LDH levels were considerably raised after I/R injury (*P*<0.001). This injury enzyme was significantly lower in the irisin-treated groups than in the IR group (*P*<0.01). The protective effect of irisin was repressed by administration of mdivi-1 (*P*=0.002). 

***Irisin activates mitochondrial function and biogenesis following I/R injury in diabetic rats***

**Figure 3:**
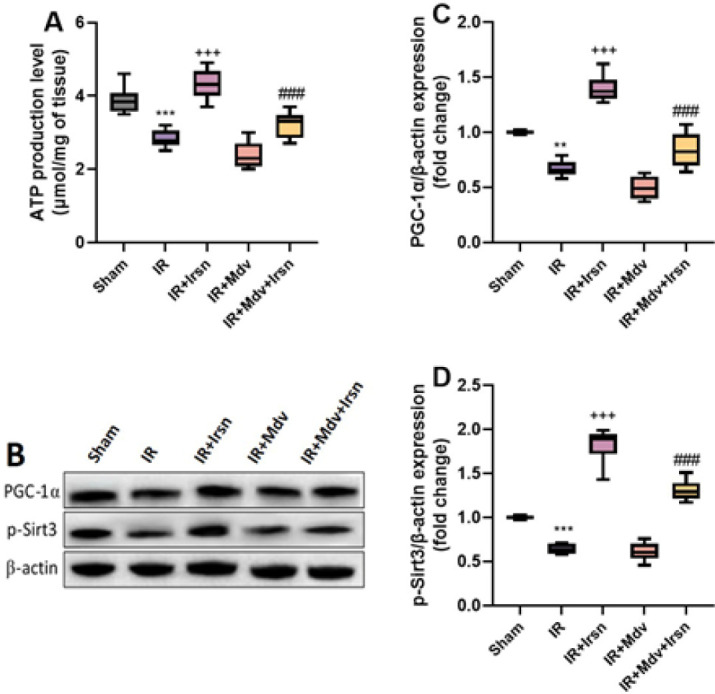
(A-D) Irisin activated mitochondrial function and biogenesis following I/R injury in diabetic rats

To investigate the potential mechanisms underlying the protective effect of irisin against I/R injury-induced arrhythmia, we examined the ATP production capacity of mitochondria and the expression of signaling molecules that participate in mitochondrial biogenesis pathways. Based on the outputs of statistical analysis with one-way ANOVA, induction of I/R injury in rats with diabetes caused reduction of ATP levels (*P*<0.001) as well as down-regulation of PGC-1α (*P*=0.002) and phosphorylated form of Sirt3 protein (p-Sirt3) compared to sham animals (*P*<0.001)(Figure 3). Irisin treatment significantly increased the ATP production level and up-regulated the PGC-1α and p-Sirt3 expression in the diabetic group who experienced I/R injury (*P*<0.001). Administration of mdivi-1 significantly suppressed the protective outcome of irisin on mitochondrial function and biogenesis markers (*P*<0.001)(Figure 3). 

***Irisin inhibits the pyroptosis pathway induced by I/R injury in diabetic rats***

**Figure 4:**
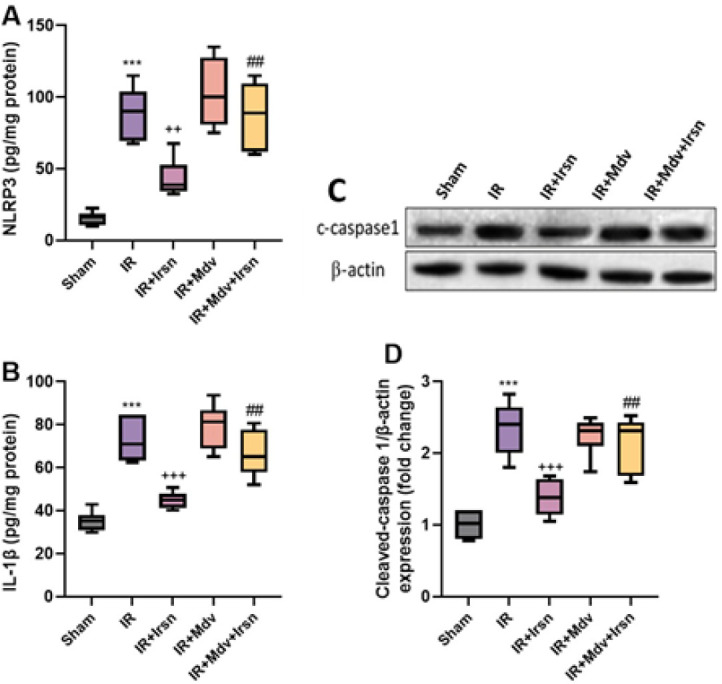
(A-D) Irisin inhibited pyroptosis pathway following I/R injury in diabetic rats

In addition, we investigated the involvement of the pyroptosis pathway in the antiarrhythmic effect of irisin against I/R injury in diabetic rats (Figure 4). One-way ANOVA revealed that the expression of pyroptosis-related proteins, NLRP3, apoptosis-associated protein caspase-1, and IL-1β was significantly increased in the diabetic rats after I/R injury (*P*<0.001), while irisin treatment significantly suppressed the expression of NLRP3 (P=0.002), cleaved caspase-1, and IL-1β (*P<*0.001). However, administration of mitochondrial division inhibitor, mdivi-1, significantly reversed the anti-pyroptotic impacts of irisin in diabetic rats with I/R injury (*P*<0.01). 

***Irisin suppresses endoplasmic reticulum stress markers following I/R injury in diabetic rats***

**Figure 5:**
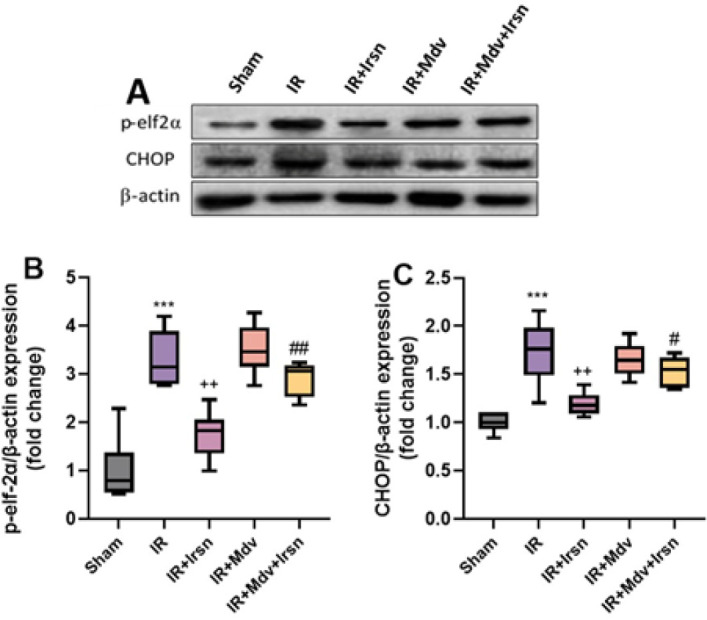
(A-C) Irisin suppressed endoplasmic reticulum stress markers following I/R injury in diabetic rats

Finally, the results of statistical comparisons using one-way ANOVA and Tukey *post-hoc* tests showed that the expression of markers of ER stress, including the phosphorylated form of eIF-2α (p-eIF-2α) and CHOP was significantly higher in the group with I/R injury when compared to the sham group (*P*<0.001), while irisin treatment significantly reduced the expression of eIF-2α (*P*=0.003) and CHOP (*P*<0.006) comparing with those of IR group. Moreover, we found that inhibition of the mitochondrial quality control system by mdivi-1 significantly up-regulated the expression of ER stress markers (p-eIF-2α, *P*=0.007 and CHOP, *P*=0.045) in diabetic rats subjected to cardiac I/R injury (Figure 5). 

## Discussion

We investigated the potential role of irisin in reducing myocardial arrhythmia and the possible mechanisms responsible for antiarrhythmic and beneficial effects of irisin in rats with diabetes in the setting of I/R injury. We found that irisin had good beneficial potential to reduce I/R injury-induced ventricular arrhythmias, and this protecting outcome of irisin was facilitated by activating the mitochondrial biogenesis and function and reducing ER stress and pyroptosis. Inhibition of mitochondrial division abolished the protective effect of irisin against myocardial injury. The results suggest that irisin may be a promising candidate for the treatment of arrhythmias following myocardial injury in diabetic patients.

Irisin is a myokine released from skeletal muscle during exercise and has been reported to have various beneficial effects on cardiovascular function (11, 12). Our study showed that irisin administration prevented the levels of LDH release from the heart and reduced the frequency and severity of cardiac arrhythmia like PVC, VT, and VF induced by I/R injury in diabetic rats. The finding suggested that irisin treatment has a protective effect against myocardial arrhythmia. Our findings demonstrated that the cardioprotective effect of irisin against I/R injury in diabetic hearts is mediated by the modulation of mitochondrial biogenesis, ER stress, and pyroptosis pathways through activating mitochondrial division, which its involvement has been reported in maintaining mitochondrial quality control and cellular homeostasis (18).

Previous studies have shown that mitochondrial biogenesis and dynamics are essential mechanisms in maintaining mitochondrial function (19). Defects in mitochondrial dynamics have a significant connection with I/R injury progress, particularly in diabetic individuals (20). Mitochondrial biogenesis is a process involving the generation of new mitochondria, which is important for maintaining cellular energy metabolism and function (19). The expression of PGC-1α and Sirt3, two key regulators of mitochondrial biogenesis, was noticeably decreased in cardiac I/R injury of diabetic rats. This result is in line with previous research that has demonstrated impaired mitochondrial biogenesis and function in diabetic hearts (21). Our data showed that irisin treatment up-regulated these mitochondrial biogenesis proteins and increased the cellular availability of ATP. These protective effects disappeared after inhibition of mitochondrial division in diabetic rat hearts. These results suggest that irisin-induced mitochondrial division could enhance mitochondrial biogenesis and quality control and prevent mitochondrial dysfunction, ultimately contributing to the reduction of myocardial arrhythmia. Activation of mitochondrial function and biogenesis can cause positive events such as saving of cell damage due to the prevention of inflammation, oxidative stress, ER stress, pyroptosis, and apoptosis (22), all of which can play a special role in reducing I/R-induced arrhythmia.

ER stress is a critical cellular response in I/R injury, involving the buildup of misfolded or unfolded proteins in the ER, leading to cellular dysfunction and apoptosis (22). CHOP is a major mediator of ER stress-induced apoptosis, and eIF-2α acts as a key regulator of ER stress in diabetic rat hearts following I/R injury (23). There was a significant increase in the expression of ER stress markers in diabetic rats with I/R injury. This confirms the previous findings demonstrating the increased ER stress in diabetic hearts (24). However, treatment with irisin substantially reduced CHOP and eIf-2α expression, suggesting that irisin may attenuate ER stress to protect against myocardial arrhythmia and promote cell survival. In addition, inhibition of mitochondrial function by Mdivi-1 not only suppressed the protective effects of irisin on ventricular arrhythmia and mitochondrial biogenesis but also abolished its positive effect on the expression of ER stress proteins. These findings support that the cardioprotective and antiarrhythmic effects of irisin are achieved in part by the promotion of beneficial interactions between mitochondria and ER in diabetic hearts. 

Moreover, pyroptosis is a type of cell death that is activated by inflammasomes and involves the initiation of inflammatory responses (4). Inflammasomes refer to intracellular multiprotein complexes that stimulate caspase-1 activation and prompt the release of pro-inflammatory cytokines like IL-1β, leading to the induction of inflammation (4, 5). Our findings showed that there was a significant increase in pyroptosis in diabetic rats with I/R injury, consistent with previous studies that have established a link between pyroptosis and myocardial reperfusion injury (25). However, treatment with irisin significantly inhibited pyroptosis, as evidenced by down-regulation of NLRP3 inflammasome, IL-1β, and cleaved caspase-1, suggesting that irisin may protect against myocardial arrhythmia by inhibiting pyroptosis and pyroptosis-induced inflammation and cell death. Importantly, inhibition of I/R-induced pyroptosis by irisin was associated with enhancement of mitochondrial function and dynamics, and this association indicates that normal mitochondrial function is necessary to prevent the occurrence of pyroptosis and inflammatory events in the diabetic heart. However, the contribution of other I/R injury players, including cellular oxidative status and the pro-survival protein kinases of reperfusion injury salvage kinase (RISK) and survivor activating factor enhancement (SAFE) pathways (26) in irisin-induced cardioprotection could be significant and warrants further investigation in future studies.

***Limitations of the study***

Exploring a range of irisin dosages and administration frequencies could provide a deeper understanding of potential dose-dependent effects and optimal treatment strategies. Also, this study primarily focused on specific pathways in diabetes, and broadening the scope to include multiple molecular pathways could offer a more comprehensive understanding of irisin’s effects. Additionally, extending the study duration would shed light on the long-term effects and sustainability of irisin treatment. Lastly, while the study provides promising insights, transitioning these findings to clinical practice would require further research, including investigating the effects in different genders and age groups, as well as human trials, to establish safety, efficacy, and optimal treatment protocols.

A: number of premature ventricular complexes; B: number of ventricular tachycardia; C: number of ventricular fibrillation; and D: ventricular arrhythmia severity scoring. The n number umwas 6 for each group

**P<0.01, ***P<0.001 vs Sham group; ^+^*P<*0.05, ^+++^*P<*0.001 vs IR group; ^#^*P<*0.05, ^##^*P<*0.01, ^###^*P<*0.001 vs IR+Irsn group

IR: ischemia/reperfusion; Irsn: irisin; Mdv: Mdivi-1

The value of n for each group was 6. ***P<0.001 vs Sham group; ^+++^*P<*0.001 vs IR group; ^##^*P<*0.01 vs IR+Irsn group

LDH: lactate dehydrogenase; IR: ischemia/reperfusion; Irsn: irisin; Mdv: Mdivi-1

A: ATP production level; B: representative immunoblotting; C: PGC-1α protein expression level; and D: p-Sirt3 protein expression level. The value of n for each group was 6. **P<0.01, ***P<0.001 vs Sham group; ^+++^*P<*0.001 vs IR group; ^###^*P<*0.001 vs IR+Irsn group

PGC-1α: peroxisome proliferator-activated receptor gamma coactivator 1-alpha; p-Sirt3: phosphorylated form of sirtuin3; IR: ischemia/reperfusion; Irsn: irisin; Mdv: Mdivi-1

A: NLRP3 levels; B: IL-1β levels; C: representative immunoblotting for cleaved-caspase 1; and D: cleaved-caspase 1 protein expression level. The value of n for each group was 6. ***P<0.001 vs Sham group; ^++^*P<*0.01, ^+++^*P<*0.001 vs IR group; ^##^*P<*0.01 vs IR+Irsn group

NLRP3: nucleotide-binding domain, leucine-rich containing family, pyrin domain containing-3; IL-1β: interleukin-1beta; c-caspase 1: cleaved-caspase 1; IR: ischemia/reperfusion; Irsn: irisin; Mdv: Mdivi-1

A: representative immunoblotting of p-eIF2α and CHOP; B: p-eIF2α protein expression level; C: CHOP protein expression level. The value of n for each group was 6. *** *P<*0.001 vs Sham group; ^++^ *P<*0.001 vs IR group; ^#^*P<*0.05, ^##^*P<*0.01 vs IR+Irsn group

p-eIF2α: phosphorylated form of eukaryotic initiation factor 2 alpha; CHOP: C/EBP homologous protein; IR: ischemia/reperfusion; Irsn: irisin; Mdv: Mdivi-1

## Conclusion

Taken together, the results of the current study suggest that irisin treatment reduces myocardial arrhythmia following I/R injury in diabetic rats by modulating mitochondria-ER interaction and inhibiting pyroptosis through activating mitochondrial dynamics. Antiarrhythmic impacts of irisin were found to be associated with improvement of mitochondrial function and biogenesis, lessening of inflammasome formation and subsequent pro-inflammatory cytokines release, and prevention of ER stress. These results offer valuable information regarding the possible use of irisin as a therapeutic option for controlling myocardial I/R injury in subjects with diabetes. Additional research is required to investigate the potential practical uses of irisin in managing and treating diabetic cardiomyopathy.

## Data Availability

The datasets used or analyzed in the current study are available from the corresponding author upon reasonable request.
